# Reproducibility assessment of a published cell shape transition analysis workflow

**DOI:** 10.1111/jmi.70143

**Published:** 2026-07-08

**Authors:** Esteban A. Miglietta, An‐Chi Luo, Agustin A. Corbat, Shao‐Chun Hsu

**Affiliations:** ^1^ Cimini Lab Imaging Platform Broad Institute of MIT & Harvard Cambridge Massachusetts USA; ^2^ Imaging Core College of Medicine National Taiwan University Taipei City Taiwan; ^3^ Facultad de Ciencias Exactas y Naturales Instituto de Física de Buenos Aires Universidad de Buenos Aires Buenos Aires Argentina

## Abstract

Image quantification is central to modern biomedical research. However, the reproducibility of image‐based studies remains a persistent challenge due to limited access to raw data and incomplete reporting of analysis workflows. To assess these issues, the Global Bioimage Analyst Society (GloBIAS) launched a coordinated effort in 2024 to examine published bioimage analysis workflows.

Here, we evaluated the reproducibility of a published workflow for analysing cell shape transitions in *Dictyostelium discoideum*. While the reported kymographs and biological conclusions could be qualitatively reproduced, a critical upstream step – cell segmentation – was insufficiently described. Reproducing the segmentation step based on the available information required extensive parameter tuning and failed to consistently capture accurate cell contours, thereby compromising downstream analysis. To evaluate whether the downstream analytical components could be reproduced when segmentation quality was improved, we adopted an alternative segmentation strategy using ilastik, a machine learning‐based image analysis tool. Using cell‐boundary coordinates extracted from ilastik‐based masks as alternative inputs for downstream analysis, we reconstructed kymographs that partially matched the published results.

Our findings demonstrate that accessible software and raw data alone are insufficient to ensure reproducibility in image‐based research. Complete reporting of image‐processing workflows, including segmentation strategies and parameter settings, is essential for the reliable reproduction of published quantitative analyses.

## 1 INTRODUCTION

Scientific progress builds upon accumulated research findings, which collectively form the foundation for advancing human knowledge. To ensure rigor and reliability, scientific studies undergo careful peer review prior to publication. Among the criteria used to evaluate scientific quality, reproducibility is a central indicator, requiring that independent researchers can access raw data and obtain consistent results using the analysis workflows described in the original study. For example, in this study, we evaluated the reproducibility of a published workflow^1^ for analysing cell shape transitions in *Dictyostelium discoideum*. However, in image‐based research, analysis workflows are often insufficiently reported.^2^ Variations in image processing steps can substantially influence quantitative outcomes, yet these steps are frequently under‐described, thereby introducing significant sources of variability.^3^

To address these challenges, initiatives such as Quality Assessment and Reproducibility for Instruments & Images in Light Microscopy (QUAREP‐LiMi) have proposed standardised checklists for reporting imaging experiments and bioimage‐analysis workflows.^4^, ^5^ In parallel, journal editors increasingly require authors to provide detailed methodological descriptions, including image acquisition, quality control, sample preparation and image processing steps, during manuscript revision.^6^

In response to the need for systematic evaluation of workflow reproducibility, the Global Bioimage Analyst Society (GloBIAS)^7^ initiated a coordinated effort in 2024 to examine representative bioimage analysis workflows. Seven workflows were selected, covering key analytical stages such as segmentation, tracking, feature extraction and machine learning‐based modelling in cell and developmental biology. As part of this initiative, we report our attempts to reproduce a workflow for analysing the shape transition between amoeboid and fan‐shape morphologies during cell motion, as reported by Moldenhawer et al.^1^ Importantly, our objective was not to evaluate the computational reproducibility of the tool itself, but rather to examine whether the image‐analysis workflow could be independently reconstructed based on the methodological information provided in the original publication.

In the original study, AmoePy served as the computational framework integrating multiple components of the image analysis pipeline, including cell segmentation, marker placement along cell contour, temporal correspondence of markers across consecutive frames for analysing shape deformation, and data visualisation using kymographs and time‐lapse videos, as well as shape dynamic simulation.^8^, ^9^, ^10^ AmoePy supports two modes of input for cell shape analysis. In the first mode, segmentation is performed internally using the modified active contour (snake) algorithm, which is applicable to both brightfield and fluorescence images.^11^, ^12^ Following segmentation, cell contours are parameterised by assigning a fixed number of markers along the boundary using normalised arc‐length coordinates. Alternatively, segmentation can be performed using external tools, with the resulting contour coordinates subsequently imported as an input file for the spatiotemporal analysis. In both modes, markers between consecutive frames are connected using the regularised contour flows method, which constrains marker trajectories to prevent topological violations, such as changes in marker ordering over time.^8^, ^10^, ^13^ Quantitative outputs are subsequently visualised through cell tracking plots, kymographs, and dynamic representations of cell shape deformation.

By reconstructing the workflow described by Moldenhawer et al.^1^ for studying spontaneous transitions between amoeboid and keratocyte‐like migration modes in *Dictyostelium discoideum*, we use this study as a case example to investigate reproducibility barriers in bioimage analysis. Our goal is to examine whether a published image analysis workflow can be independently reproduced using the information provided in the original report. Through this process, we identified critical factors affecting reproducibility and provide a broader recommendation for improving transparency and reproducibility in image‐based research.

## 2 RESULTS

### 2.1 Availability of raw image datasets and analysis tools

To reproduce the image analysis results presented in the original study, we first requested access to the raw image datasets, which were not publicly available at the time of publication.^1^ The original workflow relied on two main tools, ImageJ and AmoePy, which were used for image quantification.

ImageJ is a widely used image analysis platform supported by extensive documentation and an active user community.^14^, ^15^ In the original study, ImageJ was used to quantify the actin wave area using manually defined thresholds. This critical step – segmentation – was performed based on visual inspection. Such a manual, user‐dependent approach introduces potential bias and limits reproducibility, particularly in the absence of clearly defined criteria. Because saving thresholded masks is a straightforward function in ImageJ, providing the masks together with the original image datasets would be valuable for clarifying the selection criteria and enabling researchers in the same field to follow and replicate the analysis workflow more consistently.

AmoePy offers a focused and well‐defined scope, specifically tailored for cell shape transition analysis. It demonstrates exceptional accessibility and long‐term availability. The software is distributed through the stable repository Zenodo and is supported by a formally published protocol.^9^, ^10^ The inclusion of a concise user guide, illustrative screenshots, and example datasets greatly facilitate its usability, representing a standard that far exceeds typical bioimage analysis reports, where methodology is often underspecified. Importantly, the availability of example datasets further helps software adoption by allowing users to explore the software and become familiar with its interface. Following the provided documentation, we successfully installed and executed AmoePy using conda‐based environments on both Windows 11 and macOS Sequoia 15.6.1.^9^, ^10^

To evaluate the reproducibility of the image data analysis in the published article, we selected to reconstruct two representative kymographs from the original study that exhibit clear patterns of cell shape transition (figures 2D and 4C in the original study^1^). While the original study also quantified actin wave areas using ImageJ based on visual inspection and the original thresholding definition was not provided, this component could not be independently reproduced. As a result, our assessment focused on the AmoePy‐based workflow.

### 2.2 Reproducibility of the cell segmentation step

Because accurate cell segmentation is a prerequisite for all downstream cell shape analysis, we first attempted to reproduce this step using the built‐in snake algorithm in AmoePy. The original publication states that a modified snake algorithm was used for segmentation and notes that an implementation is available within AmoePy.^1^ However, it is not explicitly disclosed whether the implemented algorithm is identical to the one used in the original study, nor are specific parameter settings provided. Based on the available documentation, we initially inferred that the internal snake algorithm in AmoePy would be sufficient to successfully reproduce the segmentation results.

The snake algorithm (active contour model) uses a deformable curve that evolves by minimising an energy function composed of internal smoothness constraints and external image‐based forces. The external force is primarily driven by the intensity gradient between the object and the background. Strong gradients typically occur at object boundaries where intensity changes sharply, attracting the deformable curve toward edges. In contrast, the internal smoothness constraints penalise excessive stretching and bending of the curve, promoting continuity and regularity during deformation. Together, the image‐based forces guide the curve toward object boundaries, while the smoothness constraints help maintain a stable cell shape throughout the segmentation process.^11^, ^12^

We initiated segmentation using default parameters, with adjustments limited to values extracted from image metadata. Specifically, number of pixels per mm were set to 3855 and 3372 (for figures 2 and 4 of the original paper, respectively), and the time interval between frames (s), minimum length of bounding box (px), minimum area of blob (px), and cover‐up radius of removed blob (px) were initially set to 4, 15, 25, and 9, respectively. All other parameters were kept at default values, including number of segmentation points, convergence threshold, iteration numbers, and snake‐related physical parameters (elasticity, rigidity, viscosity, external force weight, and GVF regularisation coefficient). Under these conditions, the segmentation algorithm failed to consistently delineate cell contours across all images. Tracking was disrupted due to the algorithm erroneously splitting a single cell into multiple objects (Supplementary File 1). To mitigate these issues, we systematically explored the parameter space, focusing on spatial constraints such as the number of segmentation points, minimum bounding box length, minimum object area, and blob removal radius. Despite extensive parameter tuning, segmentation remained unstable. Notably, we identified that the initialisation of the snake bounding box – the starting rectangular region that defines the initial position and size of the curve before it begins to evolve toward the cell boundary – is not configurable through the graphical user interface of AmoePy, limiting control over the initial step in the segmentation process.

Because the initial bounding box of the snake algorithm cannot be enlarged, we instead adopted an alternative strategy by downscaling the image by a factor of 0.5 in Fiji (Figures 1A and 2A). This downscaling ensured that entire cell was contained within the initial snake bounding box throughout most of time series, thereby preventing tracking failures and improving contour propagation between consecutive frames. The spatial parameters were adjusted according to each dataset: for the first dataset (Figure 1A), pixels per mm = 1927, minimum bounding box length = 7 px, minimum object area = 12 px, and blob removal radius = 6 px; for the second dataset (Figure 2A), pixels per mm = 1686, while all other spatial parameters were kept identical to those used for the first dataset. Under these conditions, the snake algorithm exhibited outward expansion, indicating an imbalance between internal and external forces. We therefore adjusted key parameters governing contour evolution, including external force weight (6.0), GVF regularisation coefficient (0.01), elasticity (9.0), and rigidity (999), while keeping all other parameters at their default values. These adjustments improved the balance between the internal and external forces governing contour evolution. As a result, the snake curve propagated more closely followed the true cell boundary. However, segmentation accuracy remained suboptimal, particularly in cells exhibiting uneven fluorescence expression in the amoeboid state (Figure 1Bc,e,g) and prominent pseudopodal structures (Figure 2Ba,b,c,d).

**FIGURE 1:**
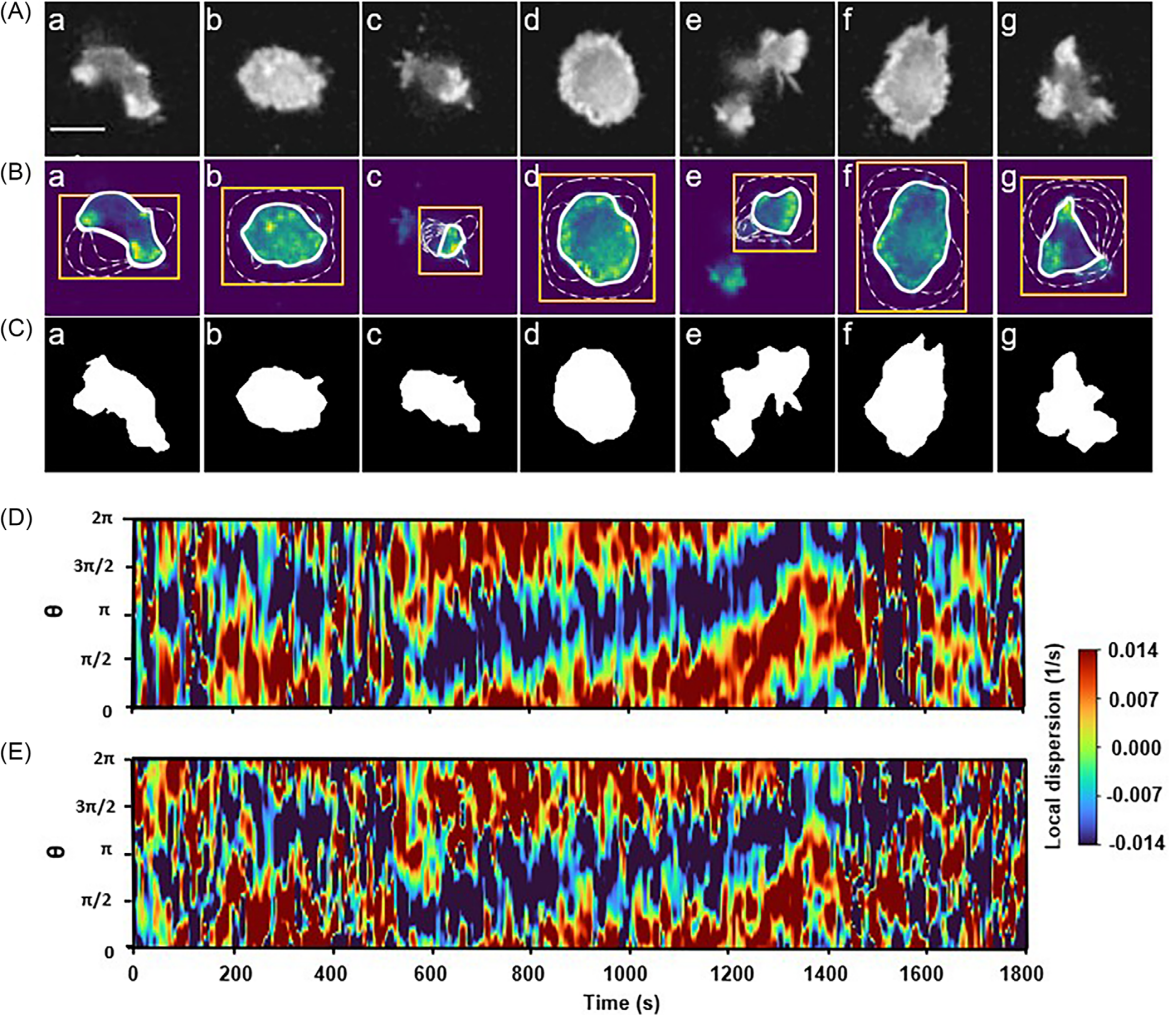
Reproduction of the reported spontaneous cell shape transition. (A) Downsized images were derived from the raw data provided by the authors under CC BY licence.^1^ Gamma and minimum display values were set to 0.28 and 60, respectively, to enhance the visibility of regions with lower fluorescence intensity. (B) Downsized images without gamma or minimum enhancement were used to extract cell contours using the segmentation function in AmoePy, showing the initial snake (orange line), intermediate contour (white dotted line) and final contour (white solid line). (C) Cell masks obtained using the ilastik‐based segmentation workflow are shown for comparison. (D) Kymograph of colour‐coded local dispersion along the cell contour generated using AmoePy and the implemented snake algorithm. The circular cell contour was parameterised into a linear format with indices from 0 to 2π, denoted as *θ* on the *y*‐axis. (E) The kymograph of the local dispersion obtained from AmoePy using the cell contour coordinates extracted from ilastik‐based segmentation (scale bar = 10 um).

**FIGURE 2:**
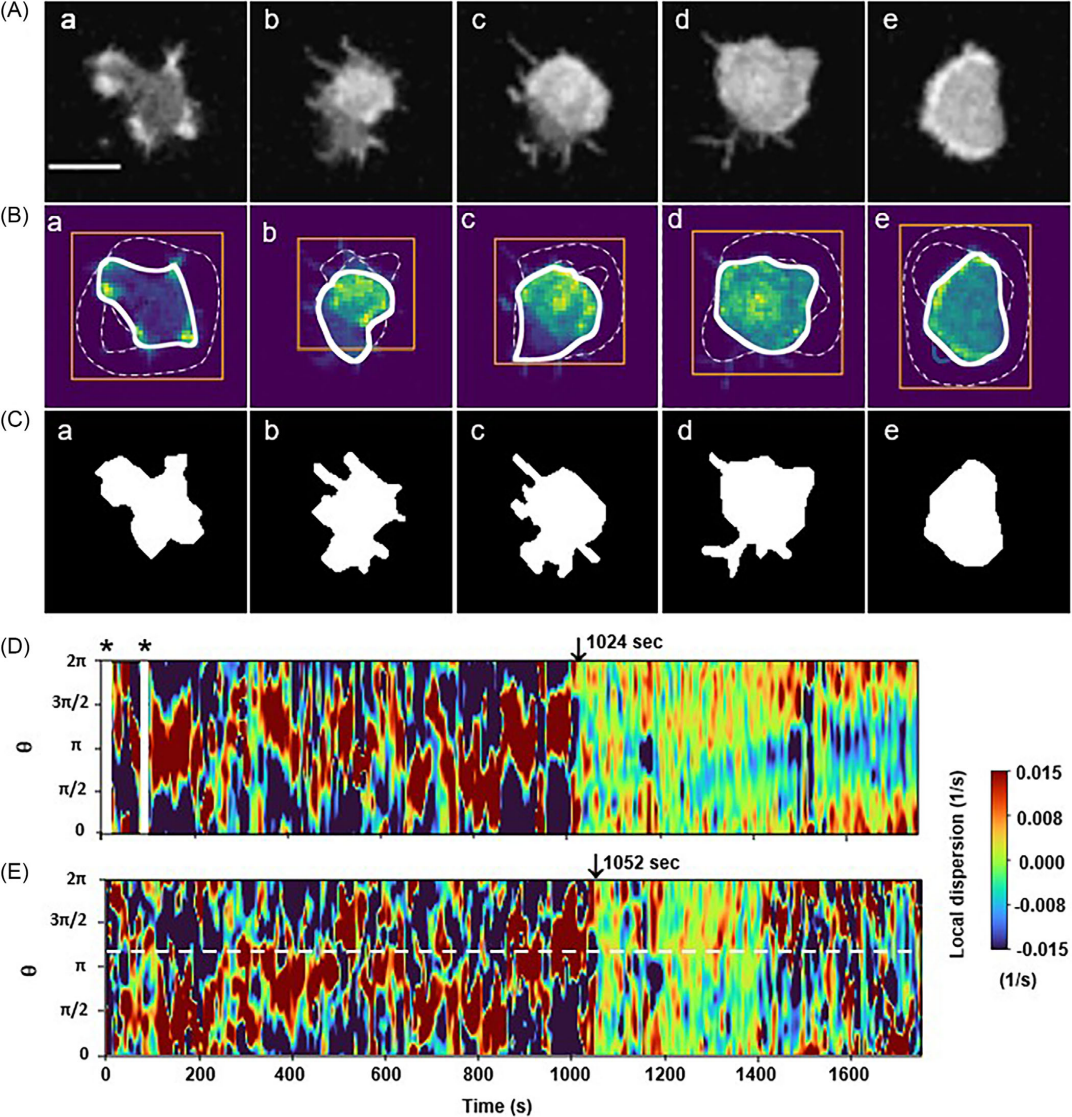
Reproduction of the reported transition from amoeboid to fan‐shaped morphology. (A) Raw images provided by the authors under a CC BY licence^1^ were downscaled by 50% using Fiji. Gamma and minimum display values were set to 0.28 and 70, respectively, to enhance the visibility of regions with lower fluorescence intensity. (B) Cell contours segmented using the internal snake algorithm implemented in AmoePy. Initial snakes are shown as orange rectangles, evolving contours as white dashed lines and final contours as white solid lines. (C) Binary masks generated using the ilastik‐based segmentation. (D) Kymograph of colour‐coded local dispersion along the parameterised cell contour using the snake algorithm. The cell was not successfully identified by the implemented snake algorithm during 0–16 and 80–100 s intervals. Therefore, these periods are shown as blank regions (*). (E) Kymograph analysed from the cell contour coordinates extracted from ilastik‐based segmentation demonstrating improved reproducibility of the original result. The dotted white line annotates the 2π position corresponding of figure 4C of the original study.^1^ Arrows indicate the onset of the fan‐shaped morphology (scale bar = 10 um).

### 2.3 Reproducibility of cell shape transition analysis under compromised segmentation quality

To quantify these shape transitions, a predefined number of virtual markers were automatically placed along the cell boundary in AmoePy. These markers function as discrete reference points that map the circular cell contour into a linear format indexed from 0 to 2π (*θ*). Temporal correspondence between markers in consecutive frames was then established using the default RegFlow settings^8^ and presented with a spatial smoothing parameter of 10. From this mapping, colour‐coded local dispersion was computed as a measurement of the local stretching rate between adjacent markers over time, enabling the identification of regions undergoing pronounced deformation during cell migration. In the resulting kymograph, positive and negative values correspond to protrusion and retraction, respectively, with protrusion shown in warm colours and retraction in cool colours.

For the first dataset, which demonstrated spontaneous switching between the amoeboid and keratocyte‐like migration modes (figure 2D in the original study^1^), the reconstructed kymograph captured cell shape transition patterns that were qualitatively similar to those reported in the original study despite segmentation inaccuracies. The principal biological finding remained reproducible: the fan‐shaped cell morphology exhibited persistent protrusion and retraction activity between 600 and 1300 s, consistent with the original study (Figure 1D vs. figure 2D ^1^). However, the local dispersion values measured from the reconstructed kymograph were noticeably higher than those reported in the original study, suggesting greater cell shape deformation in the reconstructed result. This discrepancy indicates that, although the qualitative behaviour of the cell was successfully reproduced, accurate reproduction of the quantitative measurements remained challenging.

The second dataset showed the cell can maintain a stable fan‐shaped morphology when actin wave fills the entire basal cortex (figure 4B and C in the original study^1^). Applying the same optimised parameter set yielded only partial success. Analysis could be performed only within limited temporal segments (16–80 s and 100–1751 s), requiring the dataset to be split for processing. This resulted in discontinuities in the reconstructed kymographs (Figure 2D, asterisks). Further attempts to improve segmentation through additional image downscaling led to substantial loss of spatial resolution without resolving the tracking failures. Although a period of reduced local dispersion was still detected beginning at 1024 s and persisted for approximately 420 s, indicating relative stabilisation of fan‐shaped cell, the over‐smoothed segmentation failed to preserve protrusive structures, thereby introducing analysis artefacts. Specifically, the reconstructed kymograph suggested an earlier onset of the fan‐shaped state and a longer duration of shape stabilisation compared with the analysis obtained using more accurate segmentation (Figure 2D and E, arrows). In addition, the original conclusion relied on a parallel comparison with actin wave dynamics. Because the threshold used for actin wave quantification in ImageJ was determined by visual inspection rather than an objective criterion, reproducing the analysis of actin wave was inherently challenging.

Moreover, the impact of the suboptimal segmentation was further illustrated in the curvature kymographs, in which positive (convex outward) and negative (concave inward) curvatures of the cell boundary are represented in orange and green, respectively (Figure 3). Previous studies have shown that morphological dynamics in migrating Dictyostelium cells are highly organised rather than random. This organisation is reflected by propagating curvature waves that travel along the cell boundary. Consequently, neighbouring time points in a contour‐based curvature kymograph are expected to exhibit coherent and progressively evolving patterns.^16^, ^17^, ^18^ In contrast, the curvature kymographs reconstructed using the internal snake algorithm displayed frequent discontinuities and fragmented patterns in both datasets (Figure 3A and C). These observations suggest that the contours failed to faithfully preserve the underlying spatiotemporal dynamics of the cell boundary. Because local dispersion measurements are derived directly from contour mapping, such disruption raise concerns regarding the reliability of the reconstructed deformation patterns and the quantitative interpretation of cell shape dynamics.

**FIGURE 3:**
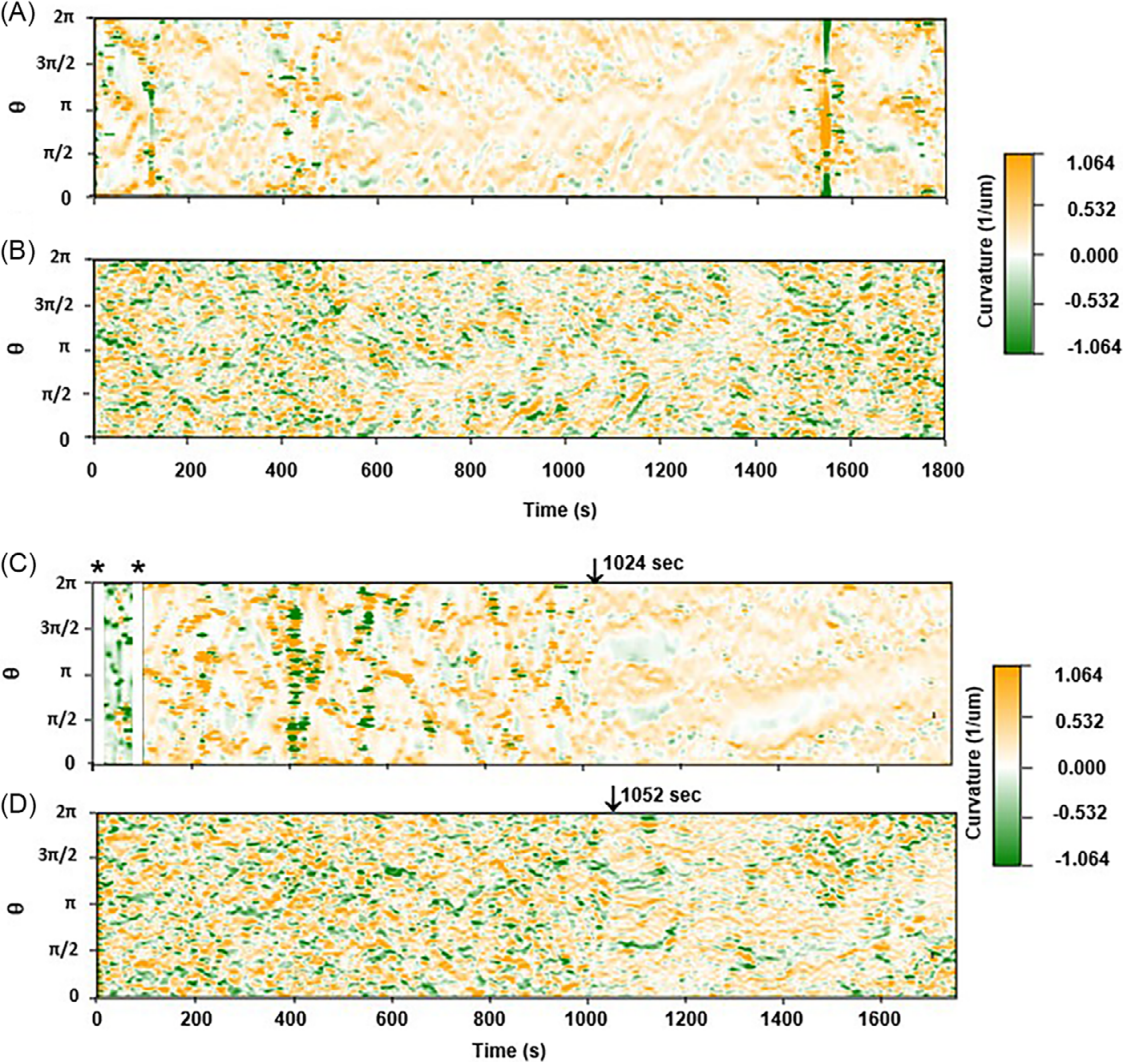
Comparison of curvature kymographs generated using snake‐ and ilastik‐based segmentation workflows. Curvature kymographs generated from the first dataset using the internal snake algorithm (A) and the ilastik‐based segmentation workflow (B). Curvature kymographs generated from the second dataset using internal snake algorithm (C) and the ilastik‐based segmentation workflow (D).

Collectively, these results demonstrate that accurate segmentation is prerequisite for reproducible cell shape transition analysis. While the major biological observations could still be qualitatively reproduced under compromised segmentation conditions, quantitative measurements and spatiotemporal features were substantially affected by contour reconstruction errors. Our findings highlight the importance of comprehensive reporting of image‐processing workflows, segmentation parameters, and analysis settings to enable faithful analysis by independent researchers. Such transparency is essential not only for reproducing the final biological conclusions but also for ensuring reproducibility of the underlying quantitative evidence on which those conclusions are based.

### 2.4 Reproducibility of cell shape transition analysis using an alternative segmentation strategy

To evaluate whether the downstream analytical components of the workflow could be reproduced, when segmentation quality was improved, we employed a machine learning–based pixel classifier in ilastik^19^ and post‐processing in Fiji to generate cell masks that more accurately captured cell boundaries (Figures 1C and 2C). Following the rational of data wrangling, the image data were subjected to Fiji for data cleaning, data transformation, data integration and followed by analysing using AmoePy for feature engineering. Non‐relevant objects were manually removed according to footprints on the maximum intensity projected image and through area‐based filtering. The cell contour coordinates at each time point were extracted from target masks and integrated in a text file which served as the input of AmoePy for analysing spatiotemporal dynamics (Figure 4).

**FIGURE 4:**
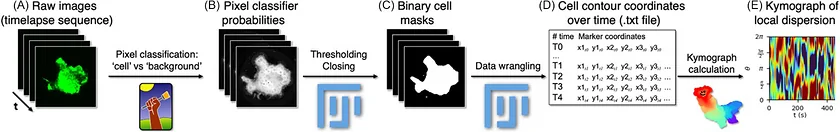
ilastik‐to‐AmoePy workflow for cell shape transition analysis. Raw images (A) were analysed using pixel classification in ilastik to generate probability maps (B), followed by image postprocessing and data wrangling macros in Fiji to generate a text file containing cell contour coordinates (D) from binary cell masks (C). These coordinates were subsequently imported into AmoePy for quantitative analysis of cell shape dynamics and generation of kymographs (E).

The cell contours were correctly captured in this workflow, particularly in cells with inhomogeneous intensity distributions and cells exhibiting prominent pseudopods (Figures 1C and 2C). Importantly, this workflow enabled robust contour tracking, as evidenced by the progressively wave‐like patterns observed in the resulting curvature kymographs (Figure 3B and D). For the first dataset, the local dispersion kymograph showed the characteristic fan‐shaped migration mode, marked by persistent protrusion and retraction activity between 600 and 1300 s (Figure 1E). For the second dataset, the ilastik‐based workflow enabled successful contour tracking throughout the entire recording period, overcoming the partial tracking failures observed with the snake algorithm (Figure 2E). Consistent with the original findings, the cell maintained a stationary fan‐shaped migration mode beginning at 1052 s (Figure 2E, arrow), while elevated local dispersion emerged at the leading edge after approximately 1400 s.

Obvious vertical phase shifts were observed (Figure 2E, white line) relative to the original publication.^1^ This discrepancy arises because AmoePy automatically assigns the coordinate origin (*θ* = 0) when parameterising the cell boundary with virtual markers. Although this automated initialisation affects the visual alignment of the kymographs, it does not alter the underlying analytical results.

To further contextualise our reproducibility assessment, we evaluated both the original publication and the ilastik‐to‐AmoePy workflow against a community‐developed checklist for reporting established image‐analysis workflows.^5^ The checklist categorises reporting criteria into three tiers – minimal (white), recommended (grey), and ideal (dark grey) (Table 1). Although the AmoePy developers provided example datasets, tutorial documentation and access to multiple software versions through a Zenodo repository to support long term accessibility,^9^, ^10^ the selected study did not satisfy several minimal reporting requirements. While the software was cited, neither its version nor key analysis parameters were reported. Additionally, the raw image data were not publicly available at the time of publication, although they were subsequently shared upon request. To demonstrate a more transparent and reproducible reporting practice, we used the ilastik‐to‐AmoePy workflow as an example that fulfils all the criteria in detail (Table 1). All software platforms, versions, and analysis settings were explicitly documented. Furthermore, all workflow components, including the ilastik project file, processed masks, image‐processing macro and data wrangling script, have been made publicly available through the GitHub repository (https://github.com/GloBIAS‐BioimageAnalysts/AmoePy_reproducibility_publication). A step‐by‐step tutorial video and description has been provided through the HereIm workflow database and YouTube (https://hereimntuic.com/portfolio/cell‐shape‐dynamics/), enabling users to reproduce the workflow and adapt it to their own datasets.

**TABLE 1:** Checklist for publication using established workflow. Reporting criteria are grouped into three categories: minimum (white), recommended (gray), and ideal (dark gray).^5^ The evaluation is based on the information provided in the present studies together with materials supplied by the software developer.

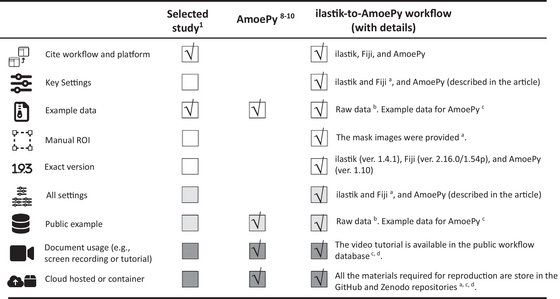

*Note*: The evaluation is based on the information provided in the study using the specific tool, as well as on the materials prepared by the tool developers.

^a^ https://github.com/GloBIAS‐BioimageAnalysts/AmoePy_reproducibility_publication

^b^ Raw datasets were kindly provided by the original authors upon request.

^c^ https://zenodo.org/records/15098663

^d^ https://hereimntuic.com/portfolio/cell‐shape‐dynamics/.

## 3 DISCUSSION

Our study highlights a fundamental challenge in image‐based research: reproducing published results is not always straightforward. Even when well established software tools are employed, reproducibility can be hindered by restricted access to original datasets, incomplete reporting of analysis criteria, and insufficient documentation of processing steps and parameter settings. In the study by Moldenhawer et al. (2022),^1^ key parameters related to actin wave thresholding, snake‐based segmentation, contour flow regularisation, and data smoothing were not documented, and the raw datasets were not publicly available at the time of publication. Although the authors generously assisted by sharing the datasets and clarifying aspects of the workflow, successful reproduction still required substantial tweaking and troubleshooting. Segmentation using AmoePy's built‐in snake algorithm was unstable at the original image resolution, whereas image downscaling combined with parameter optimisation improved performance. Alternatively, contour extraction from ilastik‐based masks enabled robust analysis and yielded more reliable results. Importantly, these challenges should not be interpreted as shortcomings of the original study alone. Rather, they reflect broader community‐wide issues concerning workflow reporting, data accessibility, and reproducibility standards in bioimage analysis. As image‐analysis pipelines become increasingly complex, comprehensive documentation of datasets, software configurations, analysis parameters, and processing workflows is essential to facilitate independent validation, workflow reuse, and long‐term reproducibility.

A key lesson is that segmentation performance depends strongly on image quality, including image resolution, contrast, signal‐to‐noise ratio, and background uniformity, all of which are often incompletely reported. Consequently, segmentation strategies that perform well on one dataset may fail when applied to another. In this case, the snake algorithm was particularly sensitive to non‐target structures in the surrounding background and to fluorescence inhomogeneity within cells. Segmentation at the original image resolution was unstable, whereas image downscaling improved performance by altering contour initialisation and reducing the influence of image noise. In contrast, ilastik provided explicit foreground–background classification, substantially improving boundary definition and contour reconstruction. These observations demonstrate that image quality is not merely a technical consideration but a critical determinant of downstream analysis outcomes. Importantly, image quality is influenced by multiple factors throughout the imaging workflow, including sample preparation, microscope setup and calibration, image acquisition parameters, and image preprocessing. However, information related to these quality‐control measures is often insufficiently reported, making it difficult to assess whether a segmentation strategy can be reliably transferred between datasets. As a result, the robustness and reusability of image‐analysis workflows remain challenging to evaluate across different experimental conditions.

In fact, the segmentation is a critical step for all image analysis workflows, yet it can be accomplished using a wide range of algorithms and software tools. Our findings suggest that reproducibility can be improved by integrating specialised tools for individual tasks rather than relying on a single software package to perform all entire analysis. In AmoePy, this flexibility is supported through an alternative input option that accepts cell contour coordinates generated by external segmentation tools. This feature enables users to combine AmoePy with segmentation approaches that are best suited to their image characteristics while retaining its downstream analytical capabilities. This modular workflow allowed each task to be performed by software specifically designed for its purpose, resulting in more reliable results. More broadly, our findings highlight the advantages of interoperable and modular bioimage analysis pipelines over monolithic end‐to‐end solutions, particularly for complex workflows in which different analytical steps have distinct computational requirements and may benefit from dedicated software implementations.

Crucially, user expertise is another important determinant of practical reproducibility. For researchers with limited computational training, reproducibility may be substantially hindered when default settings fail and successful execution requires extensive parameter search. In such cases, clear documentation, example datasets, and community support resources such as the Image.sc Forum can be invaluable for resolving ambiguities and identifying appropriate analytical settings. Continued efforts to improve software usability and knowledge sharing will therefore help lower technical barriers and promote reproducible bioimage analysis.

Our study revealed several interconnected reproducibility barriers to reproducibility, including incomplete data sharing, insufficient workflow documentation, limited transparency of software configurations, variability in image‐quality, and dependence on user expertise. Addressing these challenges requires coordinated efforts across the bioimage community.

- Researchers should share raw datasets together with key intermediate outputs and analysis parameters to facilitate independent validation and reuse.
- Software developers can improve transparency by implementing automated parameter logging, version tracking, interoperable data formats and comprehensive user documentation.
- Community initiatives can provide practical frameworks and reporting standards for workflow documentation, metadata collection and image quality assessment.
- Journal editors and reviewers could require the submission of self‐checking list for workflow reporting and image quality assessment alongside revised manuscripts to promote transparency and reproducibility.

Collectively, these efforts will help transform bioimage analysis workflows from expert‐dependent procedures into transparent and reproducible scientific practices.

## 4 MATERIALS AND METHODS

### 4.1 Test image data set

Raw image datasets for testing reproducibility of the selected study were kindly provided by the authors.^1^

### 4.2 Image processing by ilastik and Fiji

Raw images were preprocessed using an ilastik pixel classifier trained to distinguish the cell from background (Supplementary File 2). The resulting probability maps from ilastik were binarised at a probability ≥0.5 for the cell class, and then postprocessed by running a ‘Close’ EDM binary operation in Fiji (Supplementary File 2). The coordinates of cell contours were collected in a text file with a Fiji macro (Supplementary File 2) to serve as the input of AmoePy.

### 4.3 Software version

AmoePy (version 1.10) installed on Windows 11 and MacOS Sequoia 15.6.1 were used to test. The versions of Fiji and ilastik used were 2.16.0/1.54p and 1.4.1, respectively.

### 4.4 Software download

AmoePy is available through the Zenodo link.^10^ Trained ilastik pixel classifier and the Fiji macros for postprocessing and data wrangling are available in the GitHub repository (https://github.com/GloBIAS‐BioimageAnalysts/AmoePy_reproducibility_publication).

### 4.5 Video tutorial for ilastik‐to‐AmoePy workflow

The video tutorial for ilastik‐to‐AmoePy workflow is available in the public workflow database HereIm (https://hereimntuic.com/portfolio/cell‐shape‐dynamics/).

## CONFLICT OF INTEREST STATEMENT

The authors declare that they have no competing financial interests.

## Supporting information



Supporting Information

**Supplementary File 2**. ZIP archive containing all scripts used for image processing and contour extraction, including the ilastik pixel classifier (Amoeba_detector.ilp), the Fiji postprocessing macro (Ilastik_output_postProcessing.ijm), and the Fiji contour extraction macro (ExtractContourFAmoePy_20260611.ijm).
